# Neodymium Nitrate Suppresses Malignant Phenotypes in Hep-G2 Cells by Targeting Nrf2 to Inhibit EMT and Induce Ferroptosis

**DOI:** 10.3390/toxics14090831

**Published:** 2026-09-19

**Authors:** Jing Leng, Ning Wang, Ping Xiao, Xinyu Hong, Song Yu

**Affiliations:** Institute of Chemical Toxicity Appraisal, National Key Laboratory of Environmental Health Impact Assessment of Environmental New Pollutants, Shanghai Municipal Center for Disease Control and Prevention, Shanghai 200336, China; lengjing@scdc.sh.cn (J.L.); wangning@scdc.sh.cn (N.W.);

**Keywords:** neodymium nitrate, Hep-G2, EMT, ferroptosis, Nrf2

## Abstract

Hepatocellular carcinoma (HCC) is characterized by a poor prognosis, largely driven by metastasis and therapy resistance, processes closely linked to epithelial–mesenchymal transition (EMT) and ferroptosis. Neodymium nitrate (Nd(NO_3_)_3_), a predominant neodymium species, has shown antitumor potential, yet its specific effects and underlying mechanisms in HCC remain poorly understood. This study investigated whether Nd(NO_3_)_3_ exerts anti-malignant effects in Hep-G2 cells through the Nrf2–ferroptosis axis. We treated Hep-G2 cells with Nd(NO_3_)_3_ and assessed proliferation (CCK-8), migration, invasion (wound-healing and Transwell assays), EMT markers (E-cadherin, Vimentin), Nrf2 and GPx4 expression (Western blotting, qRT-PCR), and ferroptosis indicators (ROS, MDA, GSH, Fe^2+^/Fe^3+^). Low-dose Nd(NO_3_)_3_ significantly inhibited migration, invasion, and EMT-associated marker expression without affecting proliferation and promoted ferroptotic phenotypes, as evidenced by elevated ROS, MDA, and ferrous iron expression; decreased GSH levels; and downregulated GPx4 expression. Mechanistically, Nd(NO_3_)_3_ downregulated Nrf2 expression. Nrf2 overexpression reversed the suppression of migration, invasion, EMT-associated markers, and ferroptotic phenotypes, while the ferroptosis inhibitor deferoxamine rescued migration and invasion. These findings suggest that Nd(NO_3_)_3_ exerts anti-malignant effects in Hep-G2 cells by targeting Nrf2 to coordinately suppress EMT and promote ferroptosis, identifying the Nrf2–ferroptosis axis as a potential therapeutic target in HCC cells. However, these findings are based on in vitro experiments in a single Hep-G2 cell line with *n* = 3 biological replicates, which limits statistical power and generalizability; the results should therefore be interpreted as preliminary evidence requiring further validation in additional cell lines and in vivo models.

## 1. Introduction

Hepatocellular carcinoma (HCC) is the most prevalent primary liver malignancy worldwide, characterized by aggressive invasiveness, high metastatic potential, frequent recurrence, and profound chemoresistance [1,2]. Despite substantial advancements in surgical resection, local interventional therapy, molecular targeted therapy, and immunotherapy, the prognosis of patients with advanced HCC remains exceedingly poor [3,4]. Epithelial–mesenchymal transition (EMT) is a core biological process through which HCC cells acquire migratory and invasive capabilities, enabling distant metastasis, and represents one of the key factors contributing to the poor prognosis of HCC patients [3,5]. During EMT, epithelial markers are downregulated while mesenchymal markers are upregulated, leading to reduced intercellular adhesion and enhanced motility and invasiveness [5,6]. EMT activation is also closely linked to chemoresistance and the maintenance of cancer stem cell-like properties in HCC, thus functioning as a critical regulatory node in malignant disease progression [7,8].

Accumulating evidence confirms that the malignant progression of HCC is orchestrated by multiple intracellular signaling pathways, among which nuclear factor erythroid 2-related factor 2 (Nrf2), a central regulator of cellular antioxidant responses, has attracted extensive attention for its pivotal role in oxidative stress and tumorigenesis [9,10]. Under physiological conditions, Nrf2 is sequestered in the cytoplasm by Kelch-like ECH-related protein 1 (Keap1) and undergoes constitutive degradation via the ubiquitin–proteasome pathway [11]. Hyperactivated Nrf2 promotes cancer cell proliferation, EMT, invasion, metastasis and therapeutic resistance by enhancing cellular antioxidant capacity, inhibiting apoptosis, and regulating metabolic reprogramming. Importantly, Nrf2 also functions as a potent negative regulator of ferroptosis by promoting GSH synthesis and GPx4 expression [12,13]. Given that rare earth elements including neodymium have been reported to modulate intracellular oxidative stress and redox balance [14] and that Nrf2 is a master regulator of both EMT and ferroptosis, we hypothesized that Nd(NO_3_)_3_ may exert anti-malignant effects in HCC cells through modulation of the Nrf2 signaling pathway. Consequently, the Nrf2 signaling pathway is regarded as a highly promising target for tumor intervention.

In recent years, ferroptosis, an iron-dependent form of regulated cell death driven by lipid peroxidation and excessive accumulation of reactive oxygen species (ROS), has opened a new avenue for tumor therapy [14]. Distinct from apoptosis, necrosis and autophagy, ferroptosis is triggered by iron-catalyzed accumulation of intracellular lipid peroxides, which ultimately disrupts cell membrane integrity and induces cell death [12,15,16,17]. This process is tightly regulated by the intracellular antioxidant system, particularly glutathione peroxidase 4 (GPx4), a key enzyme that limits lipid peroxidation using reduced glutathione (GSH) as a cofactor [18]. A growing number of studies have demonstrated that inducing ferroptosis can effectively inhibit the growth and metastasis of multiple tumors, including those refractory to conventional therapies [19]. Importantly, Nrf2 is a potent negative regulator of ferroptosis, as it promotes GSH synthesis and enhances cellular antioxidant defense systems [20]. Therefore, targeting the Nrf2–ferroptosis axis has emerged as a novel and effective strategy for inhibiting HCC progression.

Rare earth elements and their compounds exhibit unique biological activities, with broad research prospects in antitumor therapy [21,22]. Neodymium (Nd) is a light rare earth element widely used in industry for the production of high-strength neodymium–iron–boron (NdFeB) permanent magnets, which are essential components in electric vehicles, wind turbines, consumer electronics, and medical devices. The increasing industrial application of neodymium has raised growing interest in its biological effects and potential health impacts [23]. Chemically, neodymium predominantly exists in the +3 oxidation state (Nd^3+^), which is the form present in Nd(NO_3_)_3_. While Nd^3+^ is not redox-active under physiological conditions, it can interact with biological molecules through coordination chemistry and may modulate intracellular redox homeostasis and calcium signaling, both of which are known regulators of the Keap1–Nrf2 pathway. Additionally, the nitrate counterion may contribute to biological effects through nitric oxide (NO)-related signaling, which has been linked to Nrf2 regulation. Previous studies have reported that Nd(NO_3_)_3_ can modulate apoptotic and ferroptotic processes in hepatocytes [24,25,26]. However, no study has yet investigated the role of Nd(NO_3_)_3_ in the Nrf2–ferroptosis axis in HCC cells or its effects on EMT and metastatic phenotypes. It is important to note that HCC exhibits substantial inter- and intra-tumoral heterogeneity, and findings derived from a single cell line may not fully recapitulate the diverse molecular landscapes observed in clinical HCC specimens. Therefore, in this study, we utilized the human HCC Hep-G2 cell line as an in vitro model to systematically evaluate the effects of Nd(NO_3_)_3_ and delineate the critical role of the Nrf2 signaling pathway in mediating its anti-malignant effects. Our findings provide preliminary evidence for the antitumor activity and mechanism of Nd(NO_3_)_3_ in this specific cellular context while acknowledging the necessity of future validation across multiple HCC cell lines and in vivo models before broader clinical translation.

## 2. Materials and Methods

### 2.1. Cell Lines, Chemical Treatment and Transfection

The human HCC cell line Hep-G2 was purchased from the National Collection of Authenticated Cell Cultures (NCACC), Chinese Academy of Sciences (Shanghai, China), with catalog number SCSP-510 and CSTR identifier CSTR:19375.09.3101HUMSCSP510. The cell line was authenticated by the provider via short tandem repeat (STR) profiling and was confirmed negative for mycoplasma, bacteria, and fungi upon receipt. Cells were periodically monitored for mycoplasma contamination using DAPI staining and morphological observation during the study. Cells were cultured in DMEM (Thermo Fisher Scientific, Waltham, MA, USA) supplemented with 10% fetal bovine serum (FBS) and 1% penicillin/streptomycin and maintained at 37 °C in a humidified atmosphere with 5% CO_2_. Nd(NO_3_)_3_ was obtained from Sigma-Aldrich Corp. (St. Louis, MO, USA). The pcDNA3.1-Nrf2 (NM_006164)-myc-c overexpression plasmid and the negative-control (NC) plasmid were synthesized by GuanNan Co., Ltd. (Hangzhou, China). Transient transfection was performed using Lipofectamine 3000 (Invitrogen, Carlsbad, CA, USA) according to the manufacturer’s instructions. Transfection efficiency and Nrf2 overexpression were verified by both Western blotting and qRT-PCR 48 h post-transfection. All primary antibodies for Western blotting, including anti-E-cadherin, anti-Vimentin, anti-Nrf2, anti-GPx4 and anti-GAPDH, were purchased from Beyotime Biotechnology (Shanghai, China). Assay kits for cell proliferation, oxidative stress and ferroptosis markers were also obtained from Beyotime Biotechnology (China) unless otherwise specified.

### 2.2. Cell Proliferation Assay

Cell proliferation was evaluated using the Cell Counting Kit-8 (CCK-8; Beyotime Biotechnology, China) assay. Hep-G2 cells were seeded into 96-well plates at a density of 2 × 10^4^ cells per well, allowed to adhere overnight, and treated with gradient concentrations of Nd(NO_3_)_3_ (0–10 μM) for 24, 48 and 72 h. To control for potential interference of Nd(NO_3_)_3_ with the colorimetric readout, medium-only blank wells (containing the culture medium and CCK-8 reagent without cells) were included for each Nd(NO_3_)_3_ concentration, and the absorbance of blank wells was subtracted from corresponding experimental wells. No significant absorbance interference was observed for Nd(NO_3_)_3_ at the concentrations tested. After incubation with the CCK-8 solution for 1 h at 37 °C, the absorbance at 450 nm was measured to assess cell proliferation. All experiments were performed in triplicate.

### 2.3. Cell Migration and Invasion Assays

A wound scratch assay and Transwell assays were performed to evaluate the migratory and invasive capabilities of Hep-G2 cells. For the wound scratch assay, 4 × 10^5^ cells were seeded into 6-well plates and cultured to confluence. A straight scratch was created in the cell monolayer using a sterile pipette tip, floating cells were removed by rinsing with PBS, and cells were treated with indicated concentrations of Nd(NO_3_)_3_. Wound closure was imaged at 0, 24 and 48 h using an inverted microscope for qualitative observation of the cell migration phenotype. This assay serves as a direct phenotypic observation and was not subjected to quantitative image analysis.

Transwell migration and invasion assays were performed using 24-well Transwell inserts with an 8 μm pore size (Corning, Tewksbury, MA, USA). For the migration assay, 5 × 10^4^ cells were resuspended in serum-free DMEM and added to the upper chamber, while 600 μL of complete DMEM with 10% FBS was added to the lower chamber as a chemoattractant. For the invasion assay, the upper chamber was pre-coated with 50 μL of Matrigel (BD Biosciences, San Jose, CA, USA) and incubated at 37 °C for 4 h to form a reconstituted basement membrane; then 1 × 10^5^ cells in serum-free DMEM were seeded into the upper chamber. After 24 h of treatment with Nd(NO_3_)_3_, non-migrated/non-invaded cells on the upper surface of the inserts were removed with a sterile cotton swab. Cells that migrated or invaded to the lower surface were fixed with 4% paraformaldehyde, stained with 0.4% crystal violet, and imaged using an inverted microscope equipped with a Moticam ProS5 Plus camera (Motic China Group Co., Ltd., Xiamen, China). Five randomly selected fields per insert were counted manually at 200× magnification, and the average cell number per field was calculated. All experiments were performed in triplicate and repeated at least three times independently. The Transwell assay served as the quantitative approach for measuring cell migration and invasion, complementing the qualitative wound-healing phenotypic observation.

### 2.4. Quantitative Real-Time Polymerase Chain Reaction

Total RNA was extracted from Hep-G2 cells using the Trizol reagent (Tiangen Biotech, Beijing, China) according to the manufacturer’s protocol. RNA concentration and purity were assessed using a NanoDrop spectrophotometer (Thermo Fisher Scientific, Waltham, MA, USA), with A260/A280 ratios between 1.8 and 2.0 considered acceptable. One microgram of total RNA was reverse-transcribed into cDNA using a commercial reverse transcription kit (Tiangen Biotech, Beijing, China) in a 20 μL reaction volume (42 °C for 15 min, followed by 95 °C for 3 min). qRT-PCR was performed using the SYBR Green master mix (Tiangen Biotech, Beijing, China) on an Applied Biosystems real-time PCR system. Each 20 μL reaction contained 10 μL of the 2× SYBR Green mix, 0.4 μL of each primer (10 μM), 2 μL of the cDNA template, and 7.2 μL of nuclease-free water. The amplification program was as follows: initial denaturation at 95 °C for 10 min, followed by 40 cycles of 95 °C for 15 s and 60 °C for 1 min. Melting curve analysis was performed after amplification to confirm product specificity. *GAPDH* was used as the endogenous reference gene. The primer sequences are listed in Table 1. The 2^−ΔΔCt^ method was used for relative quantification of gene expression. All reactions were performed in triplicate.

### 2.5. Western Blotting

Total cellular proteins were extracted using RIPA lysis buffer supplemented with a protease inhibitor cocktail to prevent protein degradation. Equal amounts of protein were separated by 10% SDS–polyacrylamide gel electrophoresis and transferred onto polyvinylidene difluoride (PVDF) membranes at 300 mA for 90 min at 4 °C. Membranes were blocked with 5% non-fat milk in Tris-buffered saline with Tween 20 (TBST) for 1 h at room temperature, then incubated with primary antibodies at 4 °C overnight. After washing with TBST, membranes were incubated with corresponding secondary antibodies for 2 h at room temperature. Target protein bands were visualized using an ECL substrate with the Chemi XX9 imaging system (SynGene, Cambridge, UK). GAPDH was used as the loading control.

### 2.6. Ferroptosis Marker Assays

Hep-G2 cells were seeded into 6-well plates at a density of 2 × 10^5^ cells per well and treated with Nd(NO_3_)_3_ for 24 h. Total protein concentration was determined using the BCA Protein Assay Kit for sample normalization. The levels of superoxide dismutase (SOD), malondialdehyde (MDA, a key end product of lipid peroxidation), and reduced glutathione (GSH) were measured using corresponding commercial assay kits according to the manufacturer’s protocols. Intracellular Fe^2+^/Fe^3+^ levels were assessed using the Calcein/PI Cell Viability/Cytotoxicity Assay Kit, and intracellular ROS levels were detected using the DCFH-DA fluorescent probe via fluorescence microscopy.

### 2.7. Statistical Analyses

Statistical analyses were performed using GraphPad Prism 6.0 (GraphPad Software; https://www.graphpad.com/). Data from at least three independent experiments are presented as the mean ± the standard error of the mean (SEM). The normality of data distribution was assessed using the Shapiro–Wilk test prior to parametric analysis; all datasets were found to be approximately normally distributed (*p* > 0.05). Data were also visually inspected using Q-Q plots, and outliers were assessed using Grubbs’ test; no significant outliers were detected. Comparisons between two groups were performed using two-tailed Student’s *t*-tests, and comparisons among multiple groups were performed using one-way analysis of variance (ANOVA). A *p*-value < 0.05 was considered statistically significant. We acknowledge that with *n* = 3 per group, statistical power may be limited for detecting small effect sizes; therefore, non-significant results are interpreted as ‘no significant effect observed’ rather than evidence of no effect.

## 3. Results

### 3.1. Effect of Nd(NO_3_)_3_ on Hep-G2 Cell Proliferation

**Figure 1:**
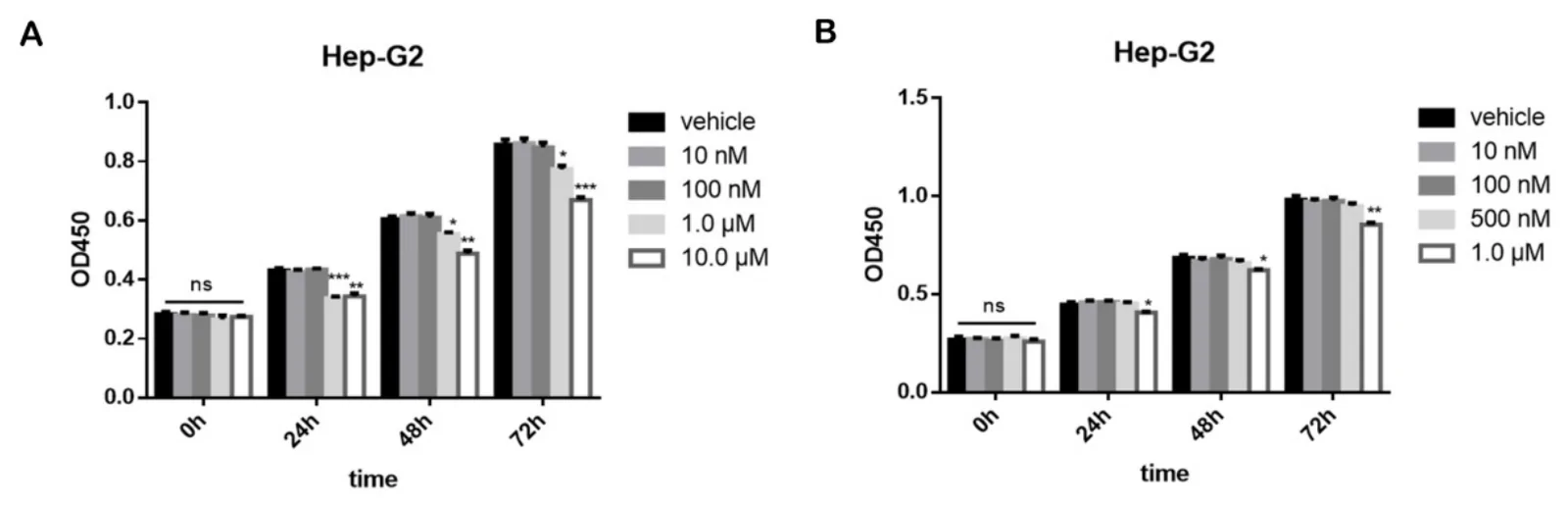
Effect of Nd(NO_3_)_3_ on Hep-G2 cell proliferation. (**A**,**B**) The impact of different doses of Nd(NO_3_)_3_ on the proliferative capacity of Hep-G2 cells was evaluated using the CCK-8 assay. ns, no statistical significance; * *p* < 0.05; ** *p* < 0.01; *** *p* < 0.001, compared with the negative control. Data were presented as the mean ± SEM of three independent experiments.

To investigate whether Nd(NO_3_)_3_ affects the proliferation of Hep-G2 cells, we treated cells with gradient concentrations of Nd(NO_3_)_3_ (0–10 μM) and assessed proliferation at 0, 24, 48, and 72 h using the Cell Counting Kit-8 (CCK-8) assay. As shown in Figure 1A, after 24 h of treatment, significant inhibitory effects on cell proliferation were observed in the 1 μM and 10 μM Nd(NO_3_)_3_-treated groups. Notably, low concentrations of Nd(NO_3_)_3_ (10–500 nM) exerted no significant effect on the proliferative activity of Hep-G2 cells compared with the control group (Figure 1B). These findings indicate that low-dose Nd(NO_3_)_3_ has no significant impact on the proliferation of Hep-G2 cells, suggesting that subsequent anti-migratory and anti-invasive effects observed at these concentrations are not attributable to cytotoxicity-mediated growth inhibition.

### 3.2. Low-Dose Nd(NO_3_)_3_ Inhibits Migration and Invasion of Hep-G2 Cells

**Figure 2:**
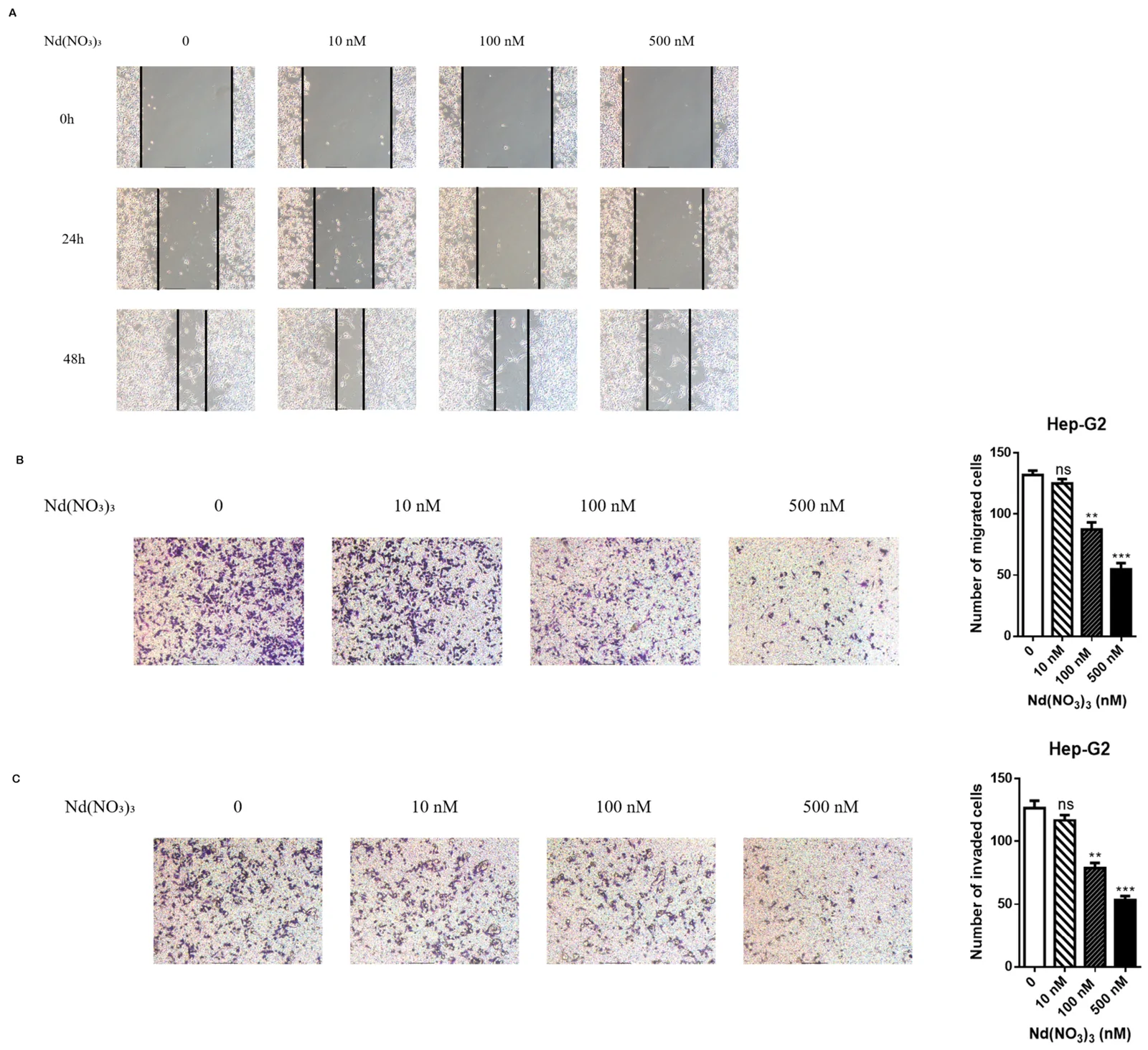
Low-dose Nd(NO_3_)_3_ inhibits migration and invasion of Hep-G2 cells. (**A**) The migration ability of Hep-G2 cells was assessed by the wound-healing scratch assay. (**B**) The Transwell migration assay was performed to evaluate the migration of Hep-G2 cells, with quantitative analysis of migrated cell numbers. (**C**) The Transwell invasion assay was conducted to determine the invasion capacity of Hep-G2 cells, with quantification of invasive cell numbers. ns, no statistical significance; ** *p* < 0.01; *** *p* < 0.001, compared with the negative control. All micrographs were captured at ×100 magnification (wound-healing assay) or ×200 magnification (Transwell assays); scale bar = 100 µm. Black lines in (**A**) mark the wound edges; purple staining in (**B**,**C**) represents crystal violet-stained cells.

To evaluate the effect of Nd(NO_3_)_3_ on the metastatic potential of Hep-G2 cells, we performed wound-healing scratch, Transwell migration, and invasion assays following treatment with low doses of Nd(NO_3_)_3_. Wound-healing scratch assay results showed that Nd(NO_3_)_3_ treatment markedly reduced the migratory ability of Hep-G2 cells in a dose-dependent manner at 24 and 48 h (Figure 2A). Consistently, the Transwell migration assay revealed that the number of migrated cells decreased significantly with increasing Nd(NO_3_)_3_ concentration (Figure 2B). The Transwell invasion assay further demonstrated that Nd(NO_3_)_3_ treatment dose-dependently reduced the number of Hep-G2 cells penetrating the Matrigel-coated membrane, indicating suppressed invasive capacity (Figure 2C). The most pronounced inhibitory effect was observed at 500 nM Nd(NO_3_)_3_, with significant differences compared with the vehicle control group (*p* < 0.01, *p* < 0.001). These results suggest that low-dose Nd(NO_3_)_3_ effectively suppresses the migratory and invasive phenotypes of Hep-G2 cells in vitro.

### 3.3. Low-Dose Nd(NO_3_)_3_ Inhibits EMT in Hep-G2 Cells

**Figure 3:**
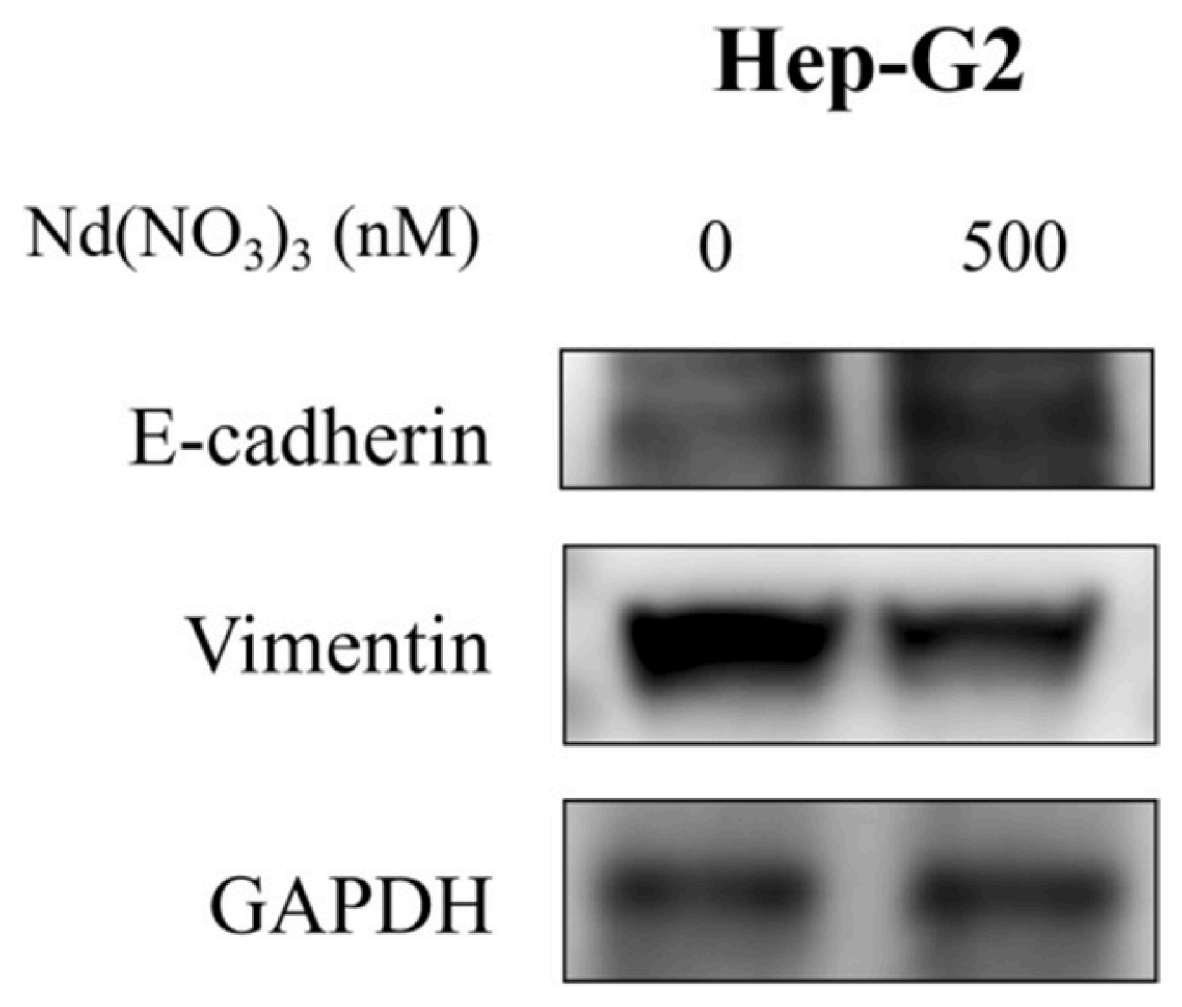
Low-dose Nd(NO_3_)_3_ inhibits EMT in Hep-G2 cells. Hep-G2 cells were treated with 500 nM Nd(NO_3_)_3_ for 24 h, and the expression levels of the EMT biomarkers E-cadherin and Vimentin were detected by Western blotting, with GAPDH used as the internal protein reference.

Given the critical role of EMT in conferring metastatic potential to HCC cells, we further examined the effect of Nd(NO_3_)_3_ on EMT-associated marker expression in Hep-G2 cells. Western blotting results showed that treatment with 500 nM Nd(NO_3_)_3_ for 24 h significantly upregulated the expression of the epithelial marker E-cadherin and downregulated the expression of the mesenchymal marker Vimentin, compared with the control group (Figure 3). These changes in E-cadherin and Vimentin expression are consistent with inhibition of EMT-like phenotypes. Collectively, these findings suggest that low-dose Nd(NO_3_)_3_ modulates EMT-associated markers in Hep-G2 cells, which may underlie its suppressive effects on cell migration and invasion. We acknowledge that additional EMT transcription factors (e.g., Snail, Slug, and Twist) should be examined in future studies to fully characterize the EMT-regulatory effects of Nd(NO_3_)_3_.

### 3.4. Nd(NO_3_)_3_ Induces Ferroptosis in Hep-G2 Cells

**Figure 4:**
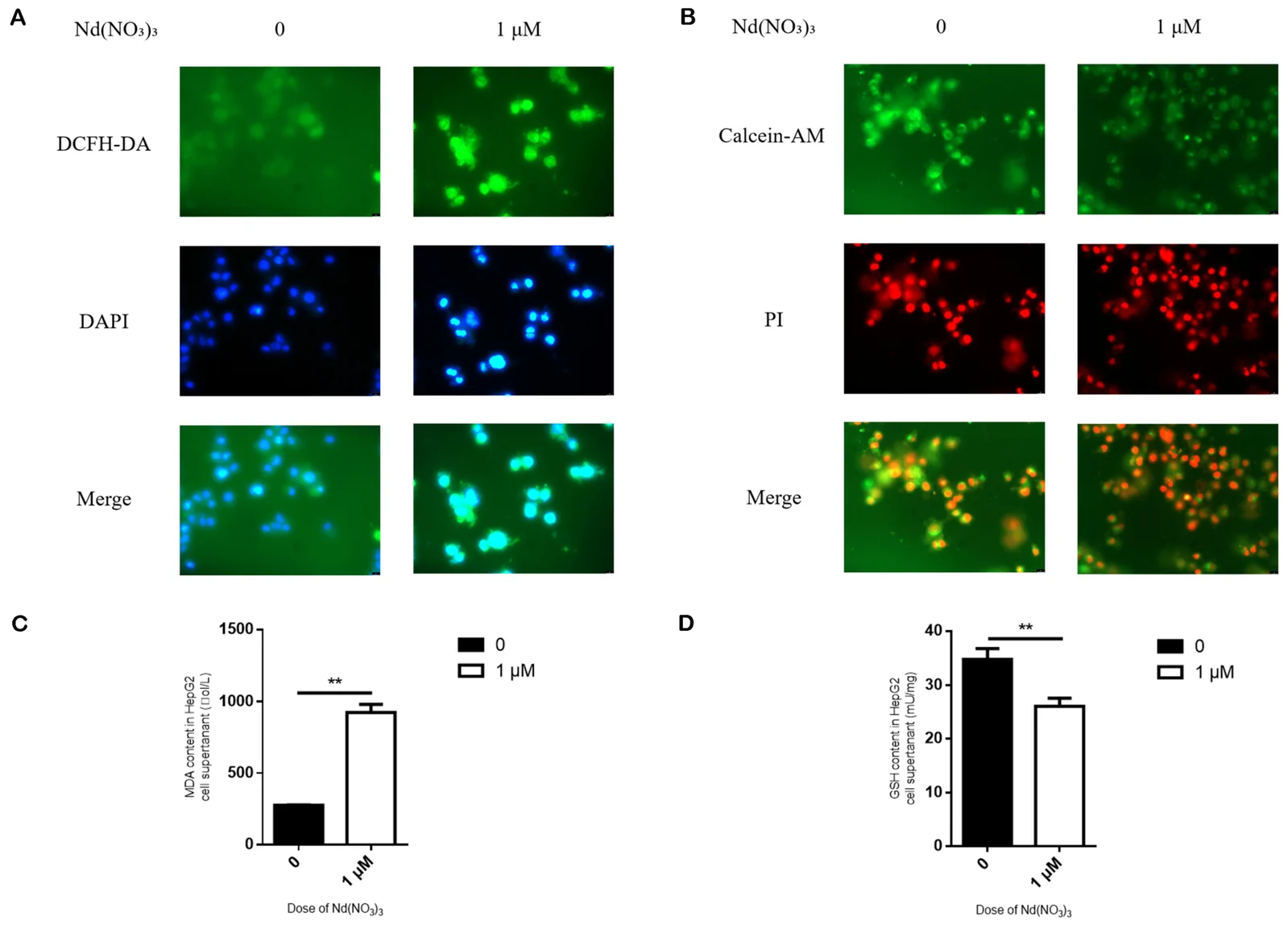
Nd(NO_3_)_3_ induces ferroptosis in Hep-G2 cells. (**A**) The ROS expression in Hep-G2 cells. (**B**) The Fe^2+^/Fe^3+^ expression in Hep-G2 cells. (**C**) The MDA expression in Hep-G2 cells. (**D**) The GSH expression in Hep-G2 cells. ** *p* < 0.01, compared with the negative control. Fluorescence micrographs were captured at ×200 magnification; scale bar = 10 µm. In (**A**), green fluorescence is the DCFH-DA channel and blue fluorescence is the DAPI channel. In (**B**), green and red fluorescence correspond to Calcein-AM and PI, respectively. Merge denotes the overlay of the corresponding channels.

To investigate whether Nd(NO_3_)_3_ regulates ferroptosis in Hep-G2 cells, we treated cells with 1 μM Nd(NO_3_)_3_ and assessed key ferroptosis markers. Fluorescence staining results showed that Nd(NO_3_)_3_ treatment significantly enhanced intracellular ROS fluorescence signals compared with the control group (Figure 4A). Calcein-AM staining further revealed a marked increase in intracellular ferrous ion levels in the Nd(NO_3_)_3_-treated group (Figure 4B), which is indicative of a ferroptotic phenotype. Biochemical assays confirmed that Nd(NO_3_)_3_ treatment significantly increased the level of MDA, a hallmark of lipid peroxidation, while markedly reducing the level of intracellular GSH in Hep-G2 cells (Figure 4C,D). Collectively, these results suggest that Nd(NO_3_)_3_ promotes ferroptotic phenotypes in Hep-G2 cells, as supported by a multi-marker panel including GSH depletion, ROS/MDA elevation, and iron accumulation. We acknowledge that direct lipid ROS measurement (e.g., via C11-BODIPY) would provide more definitive evidence for ferroptosis and should be included in future studies.

### 3.5. Ferroptosis Mediates the Inhibitory Effect of Nd(NO_3_)_3_ on Hep-G2 Cell Migration and Invasion

**Figure 5:**
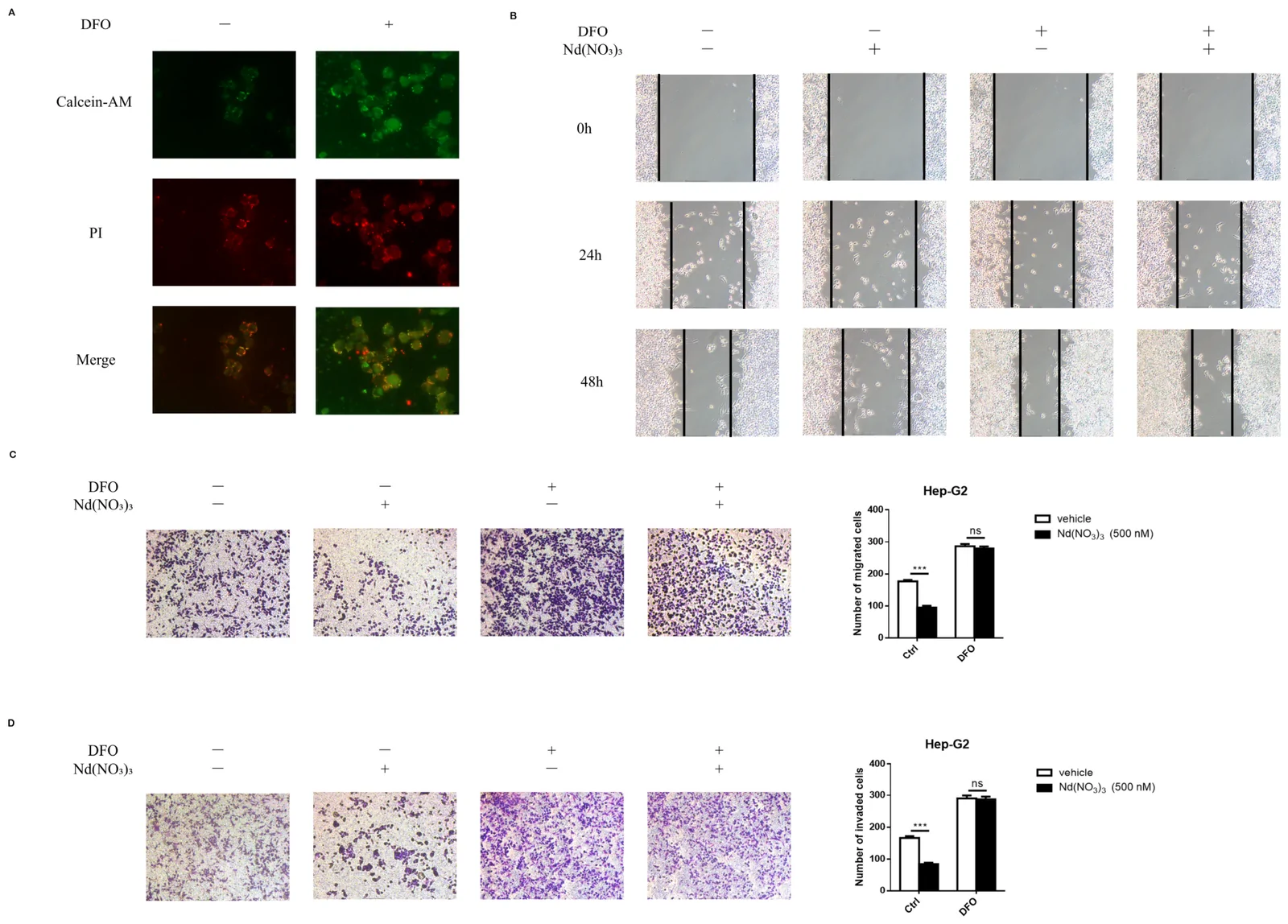
Ferroptosis mediates the inhibitory effect of Nd(NO_3_)_3_ on Hep-G2 cell migration and invasion. (**A**) The Fe^2+^/Fe^3+^ expression in Hep-G2 cells. (**B**) The migration ability of Hep-G2 cells was assessed by the wound-healing scratch assay. (**C**) The Transwell migration assay was performed to evaluate the migration of Hep-G2 cells, with quantitative analysis of migrated cell numbers. (**D**) The Transwell invasion assay was conducted to determine the invasion capacity of Hep-G2 cells, with quantification of invasive cell numbers. ns, no statistical significance; *** *p* < 0.001, compared with the negative control. Micrographs were captured at ×100 magnification (wound-healing assay) or ×200 magnification (Transwell assays); scale bar = 100 µm. Fluorescence micrographs were captured at ×200 magnification; scale bar = 10 µm. In (**A**), green and red fluorescence correspond to Calcein-AM and PI, respectively; Merge denotes their overlay. Black lines in (**B**) mark the wound edges; purple staining in (**C**,**D**) represents crystal violet-stained cells. The + and − symbols indicate the presence and absence of the indicated treatment, respectively.

To explore whether ferroptosis activation contributes to the anti-metastatic effect of Nd(NO_3_)_3_ in Hep-G2 cells, we pretreated cells with deferoxamine (DFO, 100 μM), a specific ferroptosis inhibitor, for 2 h followed by Nd(NO_3_)_3_ treatment (Figure 5A). Wound-healing scratch assay results showed that treatment with 500 nM Nd(NO_3_)_3_ alone significantly inhibited the wound-healing capacity of Hep-G2 cells, while this inhibitory effect was significantly reversed by DFO pretreatment (Figure 5B). Consistently, quantitative analysis of Transwell migration and invasion assays revealed that DFO pretreatment markedly rescued the migration and invasion of Hep-G2 cells suppressed by Nd(NO_3_)_3_, as shown by the significant recovery of migrated and invaded cell numbers in the DFO + Nd(NO_3_)_3_ group compared with the Nd(NO_3_)_3_-alone group (Figure 5C,D). These findings demonstrate that ferroptosis activation contributes to the suppressive effects of Nd(NO_3_)_3_ on Hep-G2 cell migration and invasion and that Nd(NO_3_)_3_ exerts its anti-metastatic activity at least partially in this cell line by promoting ferroptosis.

### 3.6. *Nrf2* Mediates the Inhibitory Effect of Nd(NO_3_)_3_ on Hep-G2 Cell Migration, Invasion, and EMT

**Figure 6:**
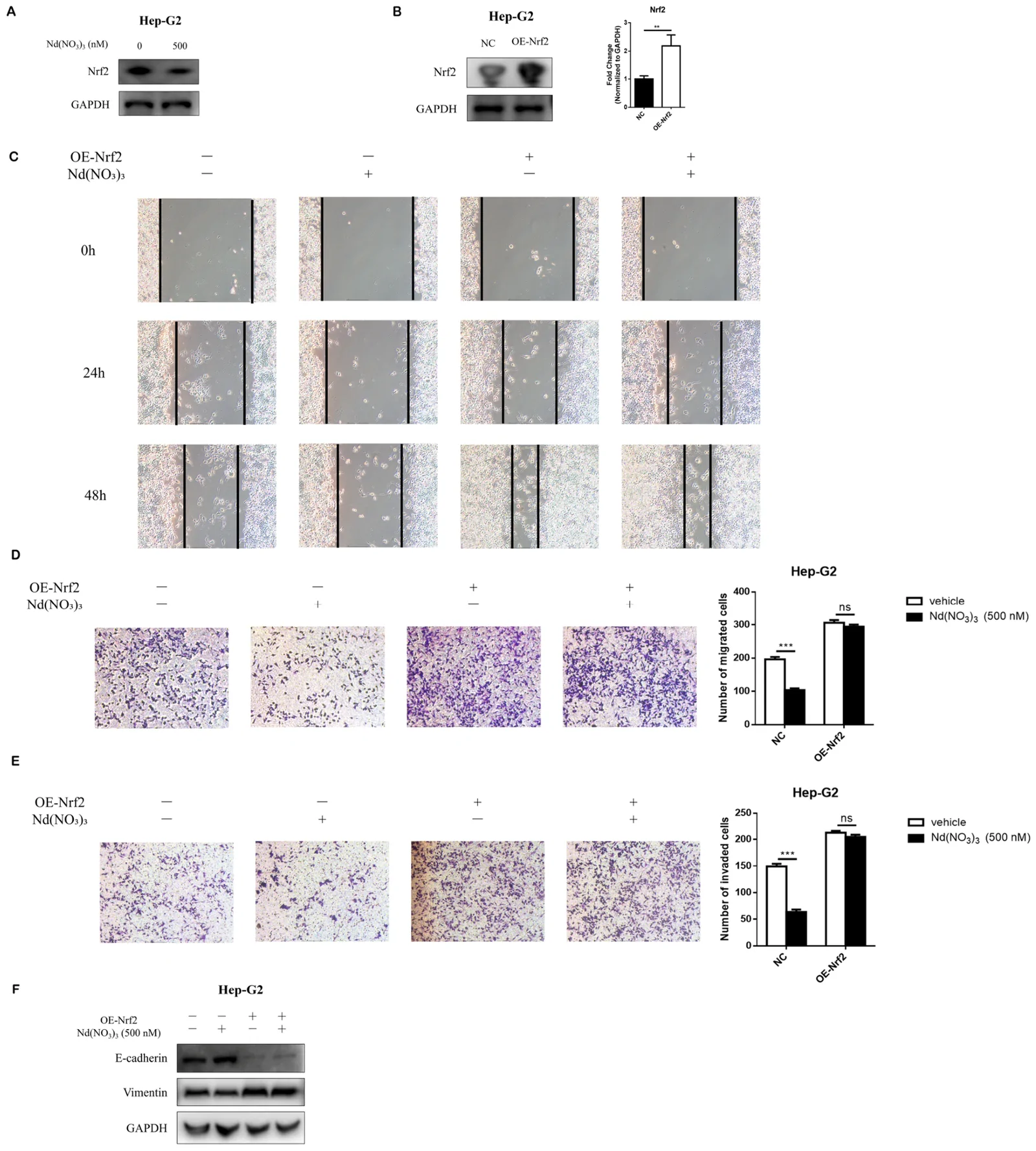
Nrf2 mediates the inhibitory effect of Nd(NO_3_)_3_ on Hep-G2 cell migration, invasion, and EMT. (**A**) Western blot analysis of Nrf2 expression in Hep-G2 cells. (**B**) Western blot and qPCR analysis of Nrf2 expression in Hep-G2 cells. (**C**) The migration ability of Hep-G2 cells was assessed by the wound-healing scratch assay. (**D**) The Transwell migration assay was performed to evaluate the migration of Hep-G2 cells, with quantitative analysis of migrated cell numbers. (**E**) The Transwell invasion assay was conducted to determine the invasion capacity of Hep-G2 cells, with quantification of invasive cell numbers. (**F**) Western blot analysis of E-cadherin and Vimentin expression levels in Hep-G2 cells, with GAPDH as the internal reference. ns, no statistical significance; ** *p* < 0.01; *** *p* < 0.001, compared with the negative control. Micrographs were captured at ×100 magnification (wound-healing assay) or ×200 magnification (Transwell); scale bar = 100 µm. Black lines in (**C**) mark the wound edges; purple staining in (**D**,**E**) represents crystal violet-stained cells. The + and − symbols indicate the presence and absence of the indicated treatment or Nrf2 overexpression, respectively.

Based on the hypothesis that Nd(NO_3_)_3_ may target the Nrf2 signaling pathway, we validated the regulatory effect of Nd(NO_3_)_3_ on Nrf2 expression in Hep-G2 cells. Western blotting results showed that treatment with 500 nM Nd(NO_3_)_3_ significantly downregulated Nrf2 protein expression in Hep-G2 cells (Figure 6A). To elucidate the functional role of Nrf2 in the anti-malignant activity of Nd(NO_3_)_3_, we established an Nrf2-overexpressing Hep-G2 cell model via transient transfection of the pcDNA3.1-Nrf2 overexpression plasmid, with empty vector-transfected cells serving as the negative control (NC). Nrf2 overexpression was verified by Western blotting and qRT-PCR 48 h post-transfection (Figure 6B).

Wound-healing scratch assay results showed that 500 nM Nd(NO_3_)_3_ markedly inhibited the migration of control Hep-G2 cells, while this inhibitory effect was significantly abrogated in Nrf2-overexpressing cells (Figure 6C). Consistently, Transwell migration and invasion assays revealed that overexpression of Nrf2 significantly reversed the suppressive effect of Nd(NO_3_)_3_ on Hep-G2 cell migration and invasion (Figure 6D,E). Furthermore, Western blotting analysis demonstrated that Nd(NO_3_)_3_-induced upregulation of E-cadherin and downregulation of Vimentin were notably reversed by Nrf2 overexpression (Figure 6F). Collectively, these results confirm that Nrf2 is a key regulator in Nd(NO_3_)_3_-mediated suppression of Hep-G2 cell migration, invasion, and EMT and that the anti-metastatic effect of Nd(NO_3_)_3_ in this cell line is dependent on the downregulation of Nrf2.

### 3.7. *Nrf2* Negatively Regulates Nd(NO_3_)_3_-Induced Ferroptosis in Hep-G2 Cells

**Figure 7:**
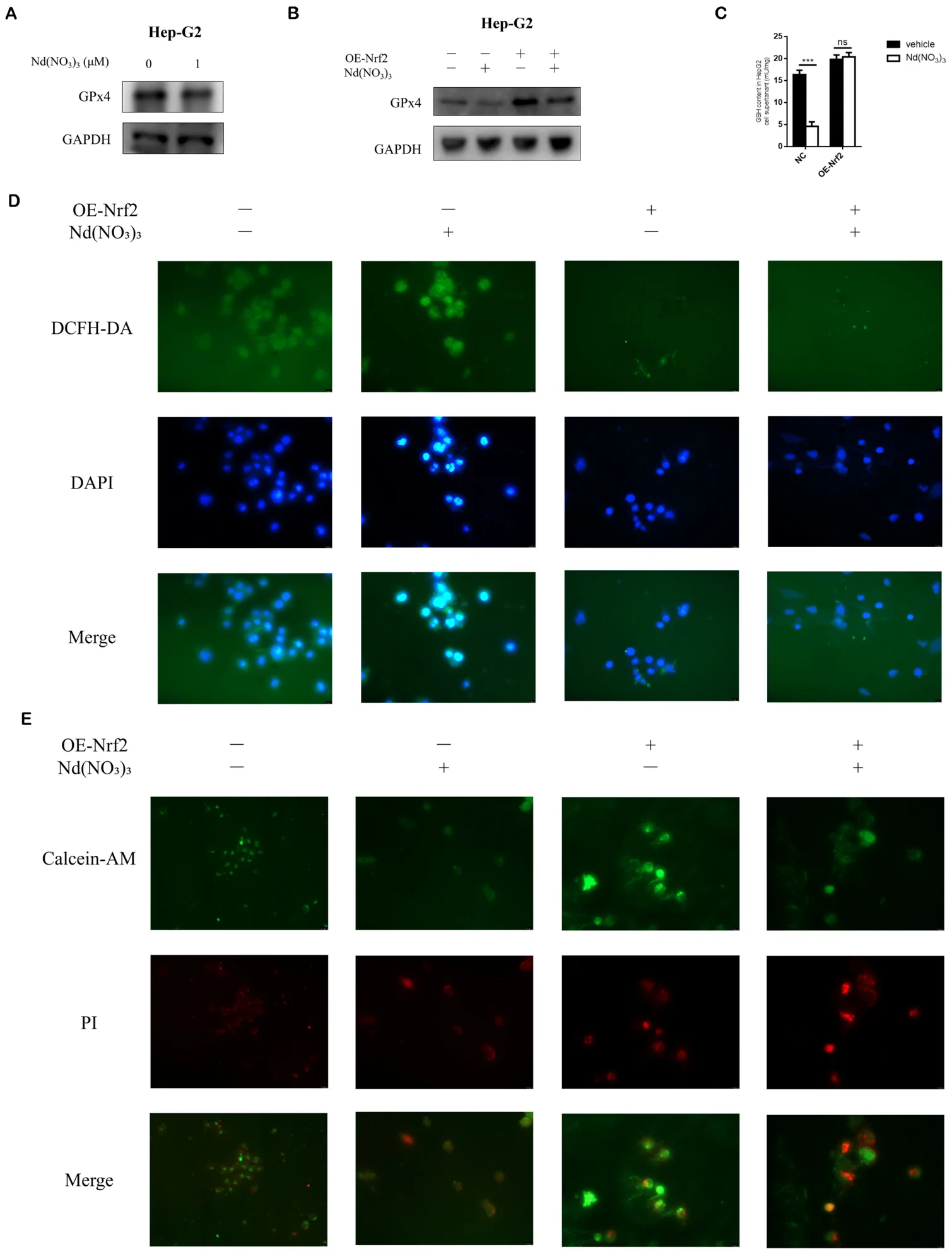
Nrf2 negatively regulates Nd(NO_3_)_3_-induced ferroptosis in Hep-G2 cells. (**A**,**B**) Western blot analysis of GPx4 expression in Hep-G2 cells. (**C**) GSH expression in Hep-G2 cells. (**D**) ROS expression in Hep-G2 cells. (**E**) Fe^2+^/Fe^3+^ expression in Hep-G2 cells. ns, no statistical significance; *** *p* < 0.001, compared with the negative control. Fluorescence micrographs were captured at ×200 magnification; scale bar = 10 µm. In (**D**), green fluorescence is the DCFH-DA channel and blue fluorescence is the DAPI channel. In (**E**), green and red fluorescence correspond to Calcein-AM and PI, respectively. Merge denotes the overlay of the corresponding channels. The + and − symbols indicate the presence and absence of the indicated treatment or Nrf2 overexpression, respectively.

As Nrf2 is a pivotal negative regulator of ferroptosis, we further investigated whether Nrf2 mediates Nd(NO_3_)_3_-induced ferroptosis in Hep-G2 cells. Western blotting results showed that the 1 μM Nd(NO_3_)_3_ treatment significantly downregulated the expression of GPx4 (Figure 7A), the key ferroptosis-inhibiting enzyme, in control Hep-G2 cells, while this effect was significantly reversed by Nrf2 overexpression (Figure 7B). Biochemical assays further revealed that the Nd(NO_3_)_3_-induced reduction in intracellular GSH levels was effectively abolished by Nrf2 overexpression, with a striking recovery of GSH content (Figure 7C).

Fluorescence staining results showed that Nd(NO_3_)_3_-induced intracellular ROS accumulation was markedly attenuated in Nrf2-overexpressing Hep-G2 cells (Figure 7D). Calcein-AM staining further confirmed that the ferroptotic phenotype induced by Nd(NO_3_)_3_ was significantly reversed by Nrf2 overexpression (Figure 7E). These findings collectively indicate that Nrf2 functions as a core negative regulator of Nd(NO_3_)_3_-induced ferroptosis in Hep-G2 cells. Nd(NO_3_)_3_ disrupts the intracellular antioxidant system by downregulating Nrf2 expression, leading to ROS accumulation, lipid peroxidation, and iron overload, thereby triggering ferroptosis in this HCC cell line.

## 4. Discussion

Rare earth elements have attracted extensive attention in the biomedical field owing to their unique physicochemical properties and biological activities. As a representative light rare earth element, neodymium has been reported to exhibit relatively good biocompatibility at low concentrations in various biological contexts; however, rare earth elements can also exhibit dose-dependent and context-dependent toxicity, particularly at higher concentrations or with prolonged exposure [27,28]. Nd(NO_3_)_3_, a common and stable neodymium compound, has been reported to inhibit proliferation, induce apoptosis, and regulate ferroptosis in hepatocytes in previous studies [25,26]. However, its exact role and underlying molecular mechanisms in HCC cell models have remained elusive until now. In this study, using the Hep-G2 cell line as an in vitro model, we demonstrated that low-dose Nd(NO_3_)_3_ exerts anti-malignant effects by targeting Nrf2 to coordinately suppress EMT and promote ferroptosis. Our findings provide preliminary evidence for the antitumor potential of Nd(NO_3_)_3_ in this specific cellular context; however, we acknowledge that these observations require validation across multiple HCC cell lines and in vivo models before broader generalization.

Accumulating evidence has demonstrated that ferroptosis not only mediates tumor cell death but is also closely associated with tumor metastatic potential. During tumor cell migration and invasion, extensive cytoskeletal remodeling and altered membrane fluidity occur, making tumor cells particularly vulnerable to lipid peroxidation and oxidative stress, which are the core drivers of ferroptosis [29,30]. In this study, we found that Nd(NO_3_)_3_ promotes ferroptotic phenotypes in Hep-G2 cells, as evidenced by increased intracellular ROS and MDA levels, reduced GSH content, elevated ferrous ion levels, and downregulated GPx4 expression. We acknowledge that some of these markers (ROS and MDA) are not entirely specific to ferroptosis and may also reflect other forms of oxidative stress; however, the combination of GPx4 downregulation, GSH depletion, iron accumulation, and the rescue by the ferroptosis-specific inhibitor DFO collectively supports the ferroptosis interpretation. More importantly, we demonstrated that inhibition of ferroptosis by DFO significantly reversed the suppressive effect of Nd(NO_3_)_3_ on Hep-G2 cell migration and invasion, confirming that ferroptosis activation contributes to the anti-metastatic activity of Nd(NO_3_)_3_ in this cellular model. This finding is consistent with recent studies showing that inducing ferroptosis can effectively suppress tumor metastasis, providing a novel strategy for HCC anti-metastatic therapy; however, the generalizability of this mechanism to other HCC subtypes remains to be determined. Direct lipid peroxidation measurement (e.g., via C11-BODIPY) and mitochondrial morphological analysis would provide more definitive evidence for ferroptosis in future studies.

**Figure 8:**
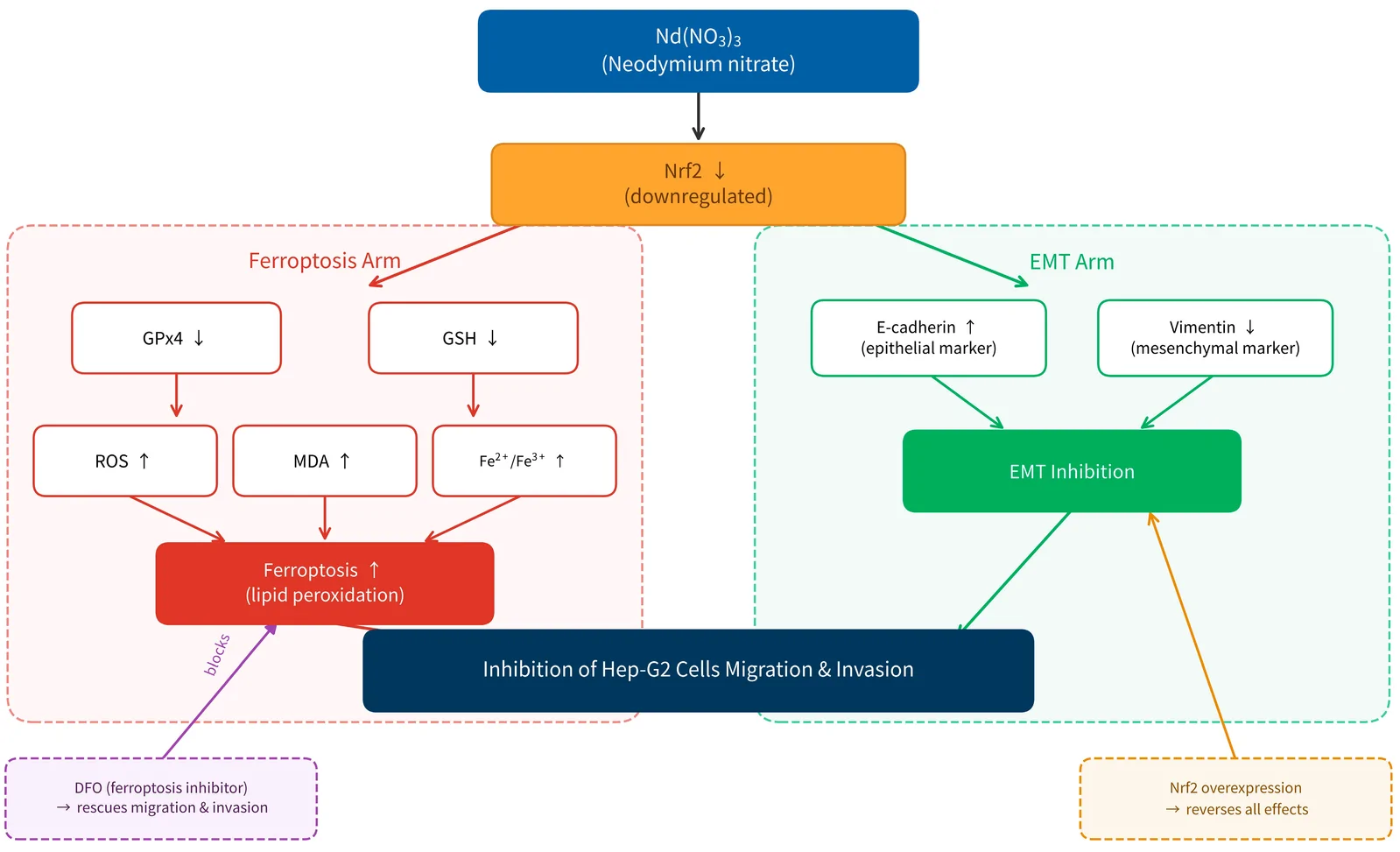
Schematic model illustrating that Nd(NO_3_)_3_ promotes ferroptosis and inhibits EMT-associated phenotypes in Hep-G2 cells by suppressing Nrf2 expression. Nrf2 downregulation leads to decreased GPx4 and GSH expression; increased ROS, MDA, and Fe^2+^/Fe^3+^ (ferroptosis arm) levels; and increased E-cadherin expression along with decreased Vimentin levels (EMT arm), collectively resulting in inhibition of HCC cell migration and invasion. Upward and downward arrows next to the markers indicate increased and decreased levels, respectively. Connecting arrows depict the proposed relationships; red and green denote the ferroptosis and EMT branches, respectively, and purple and orange denote the DFO and Nrf2-overexpression interventions, respectively.

We further elucidated the crosstalk between Nrf2, ferroptosis, and EMT in the anti-malignant activity of Nd(NO_3_)_3_ in Hep-G2 cells. Nrf2 is a master regulator of cellular redox homeostasis and functions as a potent negative regulator of ferroptosis by upregulating the expression of multiple antioxidant genes, including those involved in GSH synthesis and GPx4 expression [31,32,33]. Our results showed that overexpression of Nrf2 significantly reversed Nd(NO_3_)_3_-induced ferroptotic phenotypes in Hep-G2 cells, as evidenced by restored GSH and GPx4 levels and attenuated ROS accumulation. These findings suggest that Nd(NO_3_)_3_ promotes ferroptosis in Hep-G2 cells by downregulating Nrf2, thereby disrupting the intracellular antioxidant defense system and promoting lipid peroxidation. Notably, our study demonstrated that Nd(NO_3_)_3_ can simultaneously target two key malignant processes in Hep-G2 cells—EMT-mediated metastasis and ferroptosis-mediated cell death—via a single molecular target, Nrf2 (Figure 8).

It is critical to acknowledge the limitations of this study. First, all experiments were performed exclusively in the Hep-G2 cell line in vitro, which represents only one molecular subtype of HCC and may not fully recapitulate the inter- and intra-tumoral heterogeneity of clinical HCC. Future validation should include additional HCC cell lines (particularly Nrf2-high subtypes such as Hep3B, Huh7, SNU-449, and MHCC97H), patient-derived primary cultures and xenografts, ferroptosis-competent in vivo models, and normal hepatocytes for toxicological comparison.

Second, the detailed upstream mechanisms by which Nd(NO_3_)_3_ inhibits Nrf2 expression remain to be elucidated. Nrf2 stability is primarily regulated by Keap1-mediated ubiquitination [34,35]; Nd(NO_3_)_3_ may also modulate Nrf2 through upstream signaling pathways, including p62- [36,37], AMPK-, and PI3K/AKT/GSK-3β-mediated degradation or transcriptional regulation. Further studies using co-immunoprecipitation, ubiquitination assays, and targeted pathway inhibition are needed to determine the precise mechanism.

Third, the potential interplay between HIF-1α and the Nrf2–ferroptosis axis warrants investigation. HIF-1α and Nrf2 compete for the p300/CBP coactivator, and HIF-1α regulates ferroptosis-related genes (*GPX4*, *SLC7A11*, and *ACSL4*) and iron metabolism. Given the hypoxic microenvironment of HCC, individual hypoxia tolerance may influence sensitivity to Nd(NO_3_)_3_. A low hypoxia tolerance is known to be associated with increased HIF and NF-κB expression, as well as high proinflammatory potential [38,39], which may modify the response to therapy. This should be taken into account when interpreting the results and planning future in vivo studies. Future studies should evaluate the effects of Nd(NO_3_)_3_ under hypoxic conditions and assess HIF–Nrf2 crosstalk, particularly in the context of HCC heterogeneity and variable hypoxia tolerance among tumors.

Fourth, the toxicological profile and clinical translation potential of Nd(NO_3_)_3_ require further evaluation. While rare earth elements generally show low acute toxicity at low concentrations [40,41], high-dose or prolonged exposure can cause liver, kidney, and pulmonary toxicity [42,43]. The effects on normal hepatocytes were not assessed in this study, and comprehensive toxicological and pharmacokinetic [38] studies are essential before clinical application can be considered.

Fifth, this study assessed protein-level but not mRNA-level changes for EMT and ferroptosis genes. Future qPCR analysis of a gene panel including *CDH1*, *VIM*, *SNAI1/SNAI2*, *TWIST1*, *ZEB1*, *GPX4*, *ACSL4*, *SLC7A11*, and Nrf2 target genes (*NQO1*, *HO-1*, and *GCLC*) would clarify whether the observed changes occur at the transcriptional or post-translational level.

Finally, the lack of in vivo validation represents a significant gap, as the tumor microenvironment, immune contexture, and pharmacokinetics in living organisms may substantially alter the efficacy and safety of Nd(NO_3_)_3_. Future research should prioritize multi-cell-line validation, mechanistic dissection of Nrf2 regulation, comprehensive toxicological assessment, and in vivo efficacy studies using orthotopic HCC xenograft or genetically engineered mouse models.

## 5. Conclusions

In conclusion, this study provides in vitro evidence that, in the Hep-G2 hepatocellular carcinoma cell line, the rare earth compound Nd(NO_3_)_3_ inhibits malignant progression by targeting Nrf2 to coordinately suppress EMT-associated markers and promote ferroptotic phenotypes. These findings provide preliminary proof-of-concept data for the antitumor activity of rare earth derivatives in HCC cells and suggest the Nrf2–ferroptosis axis as a promising therapeutic target in this cellular context. We emphasize that these conclusions are derived from a single cell line with *n* = 3 biological replicates, which limits statistical power and generalizability, and should be considered as preliminary evidence rather than definitive proof of clinical efficacy. Future studies should validate these findings across multiple HCC cell lines, patient-derived primary cultures, and ferroptosis-competent in vivo systems, along with comprehensive toxicological evaluation, to assess the generalizability and translational potential of Nd(NO_3_)_3_ as an anti-HCC therapeutic agent.

## Figures and Tables

**Table 1:** The list of primers for qRT-PCR.

Gene Name	Forward Primer	Reverse Primer
*Nrf2*	AGGTTGCCCACATTCCCAAA	AGTGACTGAAACGTAGCCGA
*GAPDH*	AGGTCGGTGTGAACGGATTTG	GGGGTCGTTGATGGCAACA

## Data Availability

The data generated and analyzed in this study were deposited in the Figshare database under DOI 10.6084/m9.figshare.31808422 and are available at https://doi.org/10.6084/m9.figshare.31808422.

## Abbreviations

ANOVA	Analysis of variance
CCK-8	Cell Counting Kit-8
DFO	Deferoxamine
DMEM	Dulbecco’s modified Eagle medium
EMT	Epithelial–mesenchymal transition
FBS	Fetal bovine serum
GPx4	Glutathione peroxidase 4
GSH	Reduced glutathione
HCC	Hepatocellular carcinoma
Keap1	Kelch-like ECH-associated protein 1
MDA	Malondialdehyde
NC	Negative control
Nd(NO_3_)_3_	Neodymium nitrate
Nrf2	Nuclear factor erythroid 2-related factor 2
PBS	Phosphate-buffered saline
PVDF	Polyvinylidene difluoride
qRT-PCR	Quantitative real-time polymerase chain reaction
ROS	Reactive oxygen species
SEM	Standard error of the mean
SOD	Superoxide dismutase
TBST	Tris-buffered saline with Tween 20

## References

[1] Pan Y., Zhang X., Chen C., Hu C. (2026). Network Pharmacology and Computational Study to Identify Active Components and Potential Targets of *Polygonatum sibiricum* for Hepatocellular Carcinoma Treatment. Comb. Chem. High. Throughput Screen..

[2] Yang J., Lu H., Li L. (2025). Chemokines: Orchestration of the Tumor Microenvironment and Control of Hepatocellular Carcinoma Progression. Cancer Med..

[3] Zheleznyak A., Duraiyan D., Thunuguntla P., Camacho J., Wu S., Liu J., Weber J.D., Thorek D.L.J., Strnad B.S., Fields R.C. (2026). Epithelial-Mesenchymal Transition and Stress Adaptations Underlie Yttrium-90 Resistance in Liver Cancer Cell Lines. Cancer Res. Commun..

[4] Zhu Y., Wang M., Cao J., Song H., Zhang P. (2026). Vessels encapsulating tumor clusters in hepatocellular carcinoma: A distinct metastatic pathway with diagnostic and therapeutic significance. J. Transl. Med..

[5] Zhang C.X., Huang R.Y., Sheng G., Thiery J.P. (2025). Epithelial-mesenchymal transition. Cell.

[6] Yang Y., Huang L., Zhang N., Deng Y.N., Cao X., Liang Y., Hou H., Luo Y., Yang Y., Li Q. (2024). SUMOylation of annexin A6 retards cell migration and tumor growth by suppressing RHOU/AKT1-involved EMT in hepatocellular carcinoma. Cell Commun. Signal.

[7] Zhang S., Liu S., Dong H., Jin X., Sun J., Sun J., Wu G., Li Y. (2025). CD63-high macrophage-derived exosomal miR-6876-5p promotes hepatocellular carcinoma stemness via PTEN/Akt-mediated EMT pathway. Hepatol. Commun..

[8] Liu J., Zhang M., Li J., Tian Y., Zhao J., Liu X. (2026). FOXK1 induced upregulation of KIF20A promotes hepatocellular carcinoma progression via Wnt/beta-Catenin/EMT signaling. Cell. Mol. Life Sci..

[9] Gan L., Wang W., Jiang J., Tian K., Liu W., Cao Z. (2024). Dual role of Nrf2 signaling in hepatocellular carcinoma: Promoting development, immune evasion, and therapeutic challenges. Front. Immunol..

[10] Gupta N., Dubey V., Koiri R.K. (2025). KEAP1/NRF2 Mediated Activation of Oxidative Stress in Aflatoxin B1 Induced Early and Advanced Stage of Hepatocellular Carcinoma. J. Biochem. Mol. Toxicol..

[11] Tian Y., Tang L., Wang X., Ji Y., Tu Y. (2024). Nrf2 in human cancers: Biological significance and therapeutic potential. Am. J. Cancer Res..

[12] Huang M., Li D., Xia Z., Liao S., Si W., Yuan C., Liao Y., Wu W., Jiang M., Yu X. (2025). Silencing NRF2 enhances arsenic trioxide-induced ferroptosis in hepatocellular carcinoma cells. PLoS ONE.

[13] Li R., Wang K., Hou N., Tian Y., Gong B., Tang M. (2025). Activating transcription factor 4-mediated upregulation of Heat Shock Protein Family A Member 4 promotes hepatocellular carcinoma progression through activation of Wnt/beta-catenin/EMT signaling pathway. Int. J. Biol. Macromol..

[14] Liu D., Wu X., Hu C., Zeng Y., Pang Q. (2023). Neodymium affects the generation of reactive oxygen species via GSK-3beta/Nrf2 signaling in the gill of zebrafish. Aquat. Toxicol..

[15] Zheng Z., Juan J., Jia H., Gao L. (2026). Research on the Mechanism of LncMSTRG.5686.1/let-7a-5p Regulating Cognitive Function Alteration Induced by Neodymium Nitrate Exposure During Pregnancy and Lactation in Offspring Rats and Mediating Mitochondrial Apoptosis in SH-SY5Y Cells. J. Appl. Toxicol..

[16] Wang N., Leng J., Xu J., Qian K., Zheng Z., Tao G., Xiao P., Hong X. (2025). CircRNA_1156 Attenuates Neodymium Nitrate-Induced Hepatocyte Ferroptosis by Inhibiting the ACSL4/PKCbetaII Signaling Pathway. Antioxidants.

[17] Liang D., Minikes A.M., Jiang X. (2022). Ferroptosis at the intersection of lipid metabolism and cellular signaling. Mol. Cell.

[18] Li J., Wang Z., Wei K., Liu Q., Liang L., Lin J. (2026). Decursin induces ferroptosis via the NRF2/GPX4/SLC11A2 axis and suppresses migration in hepatocellular carcinoma. Biomed. Pharmacother..

[19] Deng J., Wu Z., Ning S., Chang X., Liu J., Yu Y., Zhang M., Zhang L. (2025). Curcumin induces ferroptosis in hepatocellular carcinoma by regulating the P62-KEAP1-NRF2-signaling pathway. BMC Cancer.

[20] Dixon S.J., Olzmann J.A. (2024). The cell biology of ferroptosis. Nat. Rev. Mol. Cell Biol..

[21] Zhang C., Liu X., Jin S., Chen Y., Guo R. (2022). Ferroptosis in cancer therapy: A novel approach to reversing drug resistance. Mol. Cancer.

[22] Zhang L., Wang X. (2025). Nelfinavir triggers ferroptosis by inducing ER stress mediated downregulation of GPX4/GSH system, upregulation of NRF2/HO-1 axis, and mitochondrial impairment in hepatocellular carcinoma cells. Cell Death Discov..

[23] Wang L., ChenLiu Z., Wang D., Tang D. (2025). Cross-talks of GSH, mitochondria, RNA m6A modification, NRF2, and p53 between ferroptosis and cuproptosis in HCC: A review. Int. J. Biol. Macromol..

[24] Chen F., Deng Q., Wu Y., Wu Y., Chen J., Chen Y., Lin L., Qiu Y., Pan L., Zheng X. (2022). U-Shaped Relationship of Rare Earth Element Lanthanum and Oral Cancer Risk: A Propensity Score-Based Study in the Southeast of China. Front. Public Health.

[25] He B., Wang J., Lin J., Chen J., Zhuang Z., Hong Y., Yan L., Lin L., Shi B., Qiu Y. (2021). Association Between Rare Earth Element Cerium and the Risk of Oral Cancer: A Case-Control Study in Southeast China. Front. Public Health.

[26] Leng J., Wang N., Chang X.L., Zhang X.P., Xu J., Yang Z.L., Qian K.L., Zheng Z.Q., Tao G.H., Jia X.D. (2025). Neodymium nitrate promotes the apoptosis of mouse liver cells via Bcl2l1/Caspase 3 pathway. Toxicol. Mech. Methods.

[27] Wang N., Lu D.S., Leng J., Du W.Q., Tao G.H., Sun J.Q., Dong C., Zheng W.D., Xiao P., Chang X.L. (2025). Chemical carcinogenicity of neodymium nitrate in AML12 hepatocytes: Dose-response metabolomics and sensitive biomarker identification. Ecotoxicol. Environ. Saf..

[28] Wang N., Leng J., Han Y., Tao G., Sun J., Dong C., Qian K., Chang X., Xiao P., Hong X. (2025). RNA-Seq Analysis of Mouse Hepatocytes AML12 Exposed to Neodymium Nitrate. Toxics.

[29] Wang W., Chen X., Wei W. (2025). TRIM22 mechanism promoting KAT2A ubiquitination degradation to regulate ferroptosis in hepatocellular carcinoma cell invasion and metastasis. Histol. Histopathol..

[30] Zhao R.R., Wu J.H., Tong L.W., Li J.Y., Lu Y.S., Shao J.W. (2024). Multifunctional metal-coordinated Co-assembled carrier-free nanoplatform based on dual-drugs for ferroptosis-mediated cocktail therapy of hepatocellular carcinoma growth and metastasis. J. Colloid Interface Sci..

[31] Chen M., Pang Y., He Y., Ma Y., Tang L. (2026). Ferroptosis Inducers Combined with Copper Ionophores Aggravate Lung Cancer-Related Fatigue via GSH Depletion and FKBP5-Associated Impairment of Nrf2/HO-1 Signaling. Cells.

[32] Gholampour F., Maghsoodi A., Malekmohammad K. (2026). Desloratadine attenuates renal ischemia-reperfusion injury through activation of the Nrf2-GCL-GSH antioxidant pathway. Curr. Res. Pharmacol. Drug Discov..

[33] Jin L., Zhang Y., Xia Y., Wu Q., Yan H., Tong H., Chu M., Wen Z. (2025). Polybrominated biphenyls induce liver injury by disrupting the KEAP1/Nrf2/SLC7A11 axis leading to impaired GSH synthesis and ferroptosis in hepatocytes. Arch. Toxicol..

[34] Jia X., Eerdemutu, Ren Y., Yang S., Yang C., Ding Q., Lin Y. (2026). The KEAP1-NFE2L2/NRF2 Axis in Non-Small Cell Lung Cancer Radioresistance: Redox Homeostasis and Emerging DNA Damage Response Mechanisms. Cancer Manag. Res..

[35] Qin H., Chen R., Xie Y., Liu H., Xiao Y., Li L. (2026). Correction: *Kummerowia striata* extract protects paracetamol-induced liver injury by modulating the S1P/Nrf2/Keap1 pathway. PLoS ONE.

[36] Zhang Y., Xu W., Cheng F., Zhu J., Lu Y., Cai G., Li J., Zhang Y. (2026). LINC01607 promotes hepatocellular carcinoma progression and ferroptosis-associated therapy resistance with functional involvement of the p62-Keap1-Nrf2 pathway. Cancer Drug Resist..

[37] Pan Z., Li M., Chen B., Lu J., Wang F., Meng C., Wang X., Shao M., He Q., Wang H. (2026). Naringenin ameliorates iron overload-associated osteoporosis via Tfeb/p62/Nrf2-mediated antioxidation. Chin. J. Nat. Med..

[38] Dzhalilova D.S., Kosyreva A.M., Tsvetkov I.S., Zolotova N.A., Sentyabreva A.V., Makarova O.V. (2023). Morphofunctional Changes in the Thymus in Prepubertal Male Wistar Rats in LPS-Induced Systemic Inflammatory Response in Relation to Hypoxia Tolerance. Bull. Exp. Biol. Med..

[39] Dzhalilova D.S., Kosyreva A.M., Diatroptov M.E., Ponomarenko E.A., Tsvetkov I.S., Zolotova N.A., Mkhitarov V.A., Khochanskiy D.N., Makarova O.V. (2019). Dependence of the severity of the systemic inflammatory response on resistance to hypoxia in male Wistar rats. J. Inflamm. Res..

[40] Zhang Y., Yang B., Xie X., Feng X., Liu X., Gong L., Luo F. (2026). Rapid, Low-Cost, Low-Temperature Pyrazole-Flux Synthesis of Imide Covalent Organic Frameworks for Uranium/Rare Earth Separation. J. Am. Chem. Soc..

[41] Santillo A., Falvo S., Venditti M., Di Fiore M.M., Grillo G., Chieffi Baccari G. (2026). Rare Earth Elements in Testicular Function: Current Knowledge and Future Directions. Int. J. Mol. Sci..

[42] Zhen D., Wang Y., Zhang X., Wang S., Li Y., Deng Q., Chen S., Li L., Cai Q., Liu Y. (2026). In vivo bioimaging and sensitive detection of uranyl via a turn-on rare-earth functionalized covalent organic framework. Biosens. Bioelectron..

[43] Dupont C.A., Franzino T., Perrey L., Beuret M., Berceaux N., Billard P. (2026). Phosphatase-mediated mitigation of rare earth element toxicity to *Pseudomonas putida*. Ecotoxicol. Environ. Saf..

